# Value of Immunohistochemical Expression of Apelin, Succinate Dehydrogenase B, Chromogranin B, Human Epidermal Growth Factor Receptor-2, Contactin 4, and Succinyl-CoA Synthetase Subunit Beta in Differentiating Metastatic From Non-Metastatic Pheochromocytoma and Paraganglioma

**DOI:** 10.3389/fendo.2022.882906

**Published:** 2022-04-28

**Authors:** Yong Wang, Danlei Chen, Yingxian Pang, Xiaowen Xu, Xiao Guan, Longfei Liu

**Affiliations:** ^1^Department of Urology, Xiangya Hospital, Central South University, Changsha, China; ^2^National Clinical Research Center for Geriatric Disorders, Xiangya Hospital, Central South University, Changsha, China

**Keywords:** immunohistochemistry, metastasis, apelin, SUCLG2, pheochromocytoma, paraganglioma

## Abstract

**Objective:**

We aimed to retrospectively collect pathologically identified pheochromocytoma and paraganglioma (PPGL) tumor tissues from our center and investigate the expression of apelin and succinyl-CoA synthetase subunit beta (SUCLG2), human epidermal growth factor receptor-2 (HER2 or ERBB-2), contactin 4 (CNTN4), chromogranin B (CHGB), and succinate dehydrogenase B (SDHB) in metastatic and non-metastatic PPGLs, for exploring their roles in the diagnosis of metastatic PPGLs.

**Methods:**

A total of 369 patients with pathologically and surgically confirmed PPGLs at Xiangya Hospital, Central South University, between June 2010 and June 2020 were retrospectively included. Sixty patients—12 patients with metastatic PPGLs and 48 patients with non-metastatic PPGLs—were selected through propensity score matching (1:4) to reduce the effect of PPGL type, sex, and age. We observed and quantified the expression of apelin, SDHB, CHGB, ERBB-2, CNTN4, and SUCLG2 in paraffin-embedded samples using immunohistochemical staining.

**Results:**

No significant differences were observed between the metastatic group and non-metastatic group with respect to the expression of CNTN4 and SUCLG2. The expression of apelin, SDHB, CHGB, and ERBB-2 was significantly different between the two groups. The expression of apelin, SDHB, and CHGB was significantly lower in the metastatic group than that in the non-metastatic group (P < 0.001). ERBB-2 expression was significantly higher in the metastatic group than in the non-metastatic group (P = 0.042). Kaplan–Meier analysis revealed that patients with negative expression of apelin, SDHB, and CHGB showed significantly lower metastasis-free survival than those with positive expression. Multivariate Cox analysis revealed that SDHB and CHGB levels were independently associated with metastasis-free survival.

**Conclusion:**

The expression levels of apelin, CHGB, SDHB, and ERBB-2 may be predictive biomarkers for the diagnosis of metastatic PPGLs. Patients with negative expression of apelin, CHGB, and SDHB should be subjected to frequent postoperative follow-up procedures

## Introduction

Pheochromocytomas (PHEOs) and paragangliomas (PGLs) are rare neuroendocrine tumors originating from chromaffin cells of the adrenal medulla and sympathetic or parasympathetic paraganglion, respectively, located in extra-adrenal tissues (1). The annual incidence of pheochromocytomas and paragangliomas (PPGLs) is approximately 2–8 cases per million, 85% of which are PHEOs (1, 2). Malignancy can only be diagnosed after the presence of distant metastases in non-chromaffin tissues (3). A total of 5–20% PHEOs would finally progress to metastasis, with a relatively higher rate of 15–35% for PGLs (4–7). The prognosis of metastatic PPGLs remains poor, with an estimated 5-year survival of 50% or less (8, 9). Thus, it is of vital importance to predict metastatic transformation in the early stages for better evaluation of prognosis and timely treatment decisions. However, only a few biomarkers have demonstrated the potential to differentiate metastatic PPGLs from non-metastatic PPGLs (10–12). Overexpression of ERBB-2, known as human epidermal growth factor receptor-2 (HER2), is associated with the onset of metastatic PPGLs (10). Contactin 4 (CNTN4) is more frequently expressed in metastatic PPGLs than in non-metastatic PPGLs (11). Immunohistochemical (IHC) analyses have identified weak or no chromogranin B (CHGB) protein expression in five of six metastatic PPGLs (12). However, the use of these biomarkers seems inadequate. New biomarkers are needed to achieve a better distinction between metastatic and non-metastatic PPGLs.

Apelin serves as an endogenous ligand for the G protein-coupled receptor, angiotensin-like-receptor 1 (APJ) (13–15). Apelin signaling is widely distributed in multiple organs and plays an important role in several physiological processes, including cardiovascular regulation, angiogenesis, and energy metabolism (15–19). Apelin may contribute to angiogenesis by mediating the adenosine monophosphate-activated kinase (AMPK)/endothelial nitric oxide synthase (eNOS), phosphatidylinositol 3-kinase (PI3K)/protein kinase B (Akt)/eNOS, and hypoxia-inducible factor-1 (HIF-1)/vascular endothelial growth factor (VEGF)/VEGF receptor (VEGFR) pathways (20). The role of apelin in cancer development and progression has been elucidated in recent years. Apelin expression is associated with metastasis in lung cancer, hepatocellular carcinoma, prostate cancer, and bladder cancer (21–24).

Succinyl-CoA synthetase subunit beta (SUCLG2) is encoded by *SUCLG2* on chromosome 3. It participates in the tricarboxylic acid (TCA) cycle by coupling succinyl-CoA hydrolysis to guanosine triphosphate (GTP) synthesis, mediating substrate-level phosphorylation (25, 26). Studies have indicated an important role of SUCLG2 in tumors (27). A total of 20% of PPGLs or more harbor mutations in genes related to the TCA cycle. Recently, eight germline variants in the GTP-binding domain of *SUCLG2* were found in 15 patients with sporadic PPGLs, suggesting the potential role of *SUCLG2* as a new candidate prognostic gene in PPGLs (28).

Considering the role of apelin in tumor angiogenesis and metastasis, we hypothesized that apelin may also contribute to the metastatic transformation in PPGLs. The role of SUCLG2 in PPGL development and progression makes it a potential biomarker for the identification of PPGLs. However, to date, no studies have been published examining the expression of apelin and SUCLG2 in PPGLs. The roles of apelin and SUCLG2 in distinguishing metastatic from non-metastatic PPGLs remain unknown. Hence, we aimed to retrospectively collect pathologically identified PPGL tumor tissues from our center and investigate the expression of apelin and SUCLG2 along with previously studied ERBB-2, CNTN4, CHGB, and SDHB in metastatic and non-metastatic PPGLs. Furthermore, we aimed to examine the importance of these biomarkers in the diagnosis of metastatic PPGLs.

## Materials and Methods

### Patient Selection

A total of 369 patients with pathologically and surgically diagnosed PPGLs at Xiangya Hospital, Central South University, between June 2010 and June 2020, were retrospectively included. All patients were subjected to follow-ups until December 2020. Metastatic PPGLs were characterized by the presence of distant metastasis at non-chromaffin sites according to the 2017 World Health Organization (WHO) classification of endocrine tumors (29). Metastasis was identified by histological examination or imaging (including computed tomography and positron emission computed tomography). The coexistence of multiple tumors and recurrence of PPGLs in chromaffin tissues were not perceived as metastases. The exclusion criteria were as follows: 1) patients with metastasis at the first visit to our hospital (N =5), and 2) unavailability of paraffin-embedded tissue specimens (N =10). Finally, 12 patients with metastatic PPGLs and 48 patients with non-metastatic PPGLs were selected through propensity score matching (1:4) to reduce the effects of PPGL type, sex, and age. Demographic and clinical data, including sex, age, biochemical examinations, and imaging findings, were also collected. This study was approved by the Medical Ethics Committee of Xiangya Hospital, Central South University. Written informed consent was obtained from all patients.

### Immunohistochemistry

**Figure 1:**
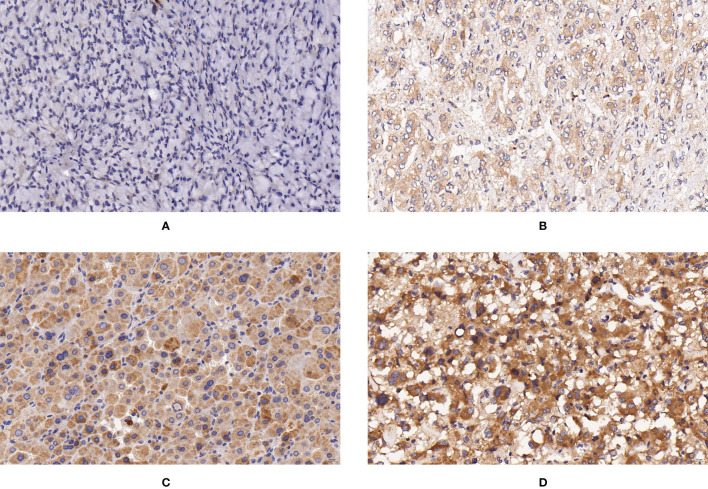
Expression of SUCLG2 in PPGLs. **(A)** Negative immunostaining of SUCLG2 in PPGLs. **(B)** Weakly positive immunostaining of SUCLG2 in PPGLs. **(C)** Intermediately positive immunostaining of SUCLG2 in PPGLs. **(D)** Strongly positive immunostaining of SUCLG2 in PPGLs.

**Figure 2:**
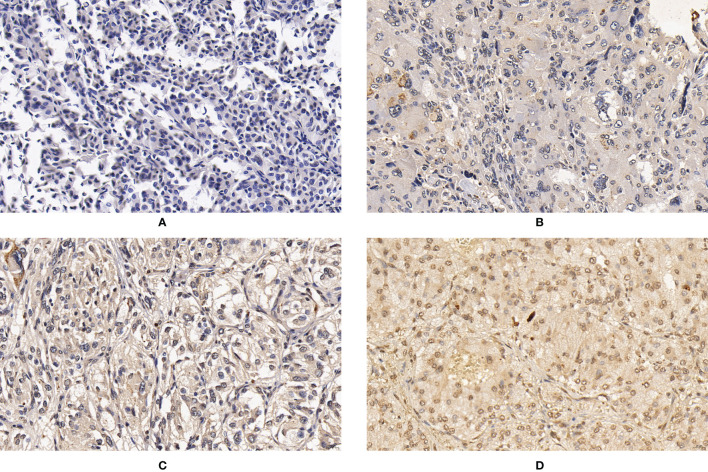
Expression of CNTN4 in PPGLs. **(A)** Negative immunostaining of CNTN4 in PPGLs. **(B)** Weakly positive immunostaining of CNTN4 in PPGLs. **(C)** Intermediately positive immunostaining of CNTN4 in PPGLs. **(D)** Strongly positive immunostaining of CNTN4 in PPGLs.

**Figure 3:**
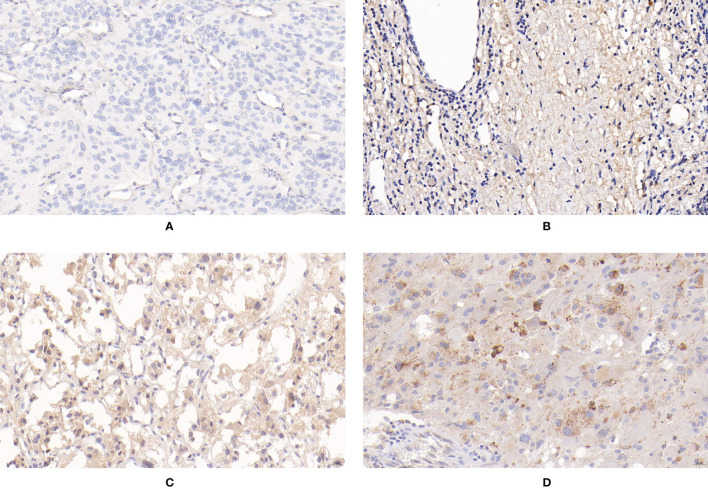
Expression of apelin in PPGLs. **(A)** Negative immunostaining of apelin in PPGLs. **(B)** Weakly positive immunostaining of apelin in PPGLs. **(C)** Intermediately positive immunostaining of apelin in PPGLs. **(D)** Strongly positive immunostaining of apelin in PPGLs.

**Figure 4:**
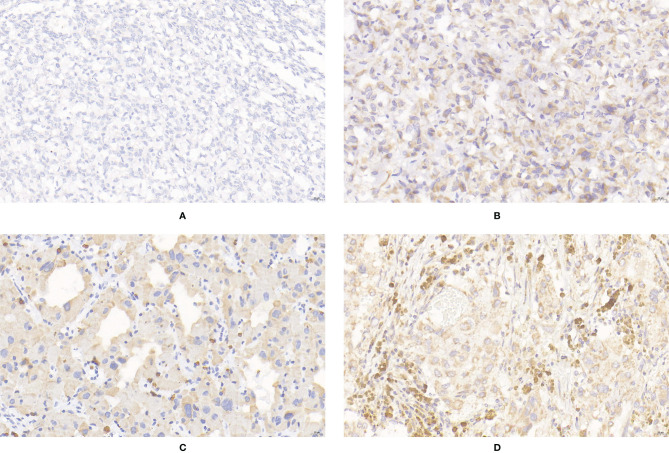
Expression of SDHB in PPGLs. **(A)** Negative immunostaining of SDHB in PPGLs. **(B)** Weakly positive immunostaining of SDHB in PPGLs. **(C)** Intermediately positive immunostaining of SDHB in PPGLs. **(D)** Strongly positive immunostaining of SDHB in PPGLs.

**Figure 5:**
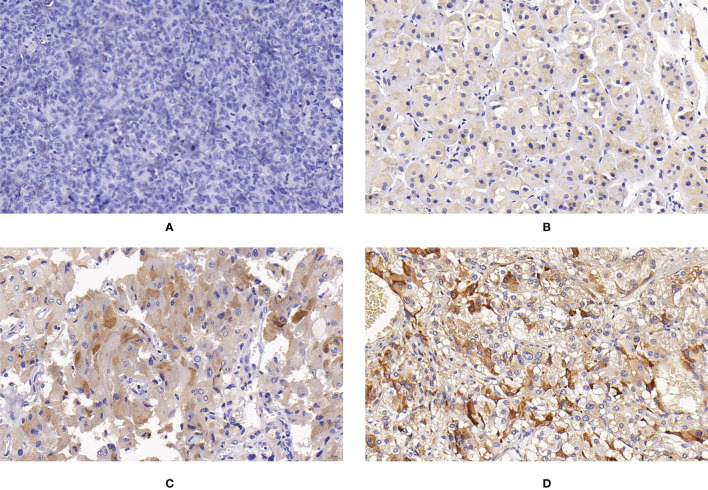
Expression of CHGB in PPGLs. **(A)** Negative immunostaining of CHGB in PPGLs. **(B)** Weakly positive immunostaining of CHGB in PPGLs. **(C)** Intermediately positive immunostaining of CHGB in PPGLs. **(D)** Strongly positive immunostaining of CHGB in PPGLs.

**Figure 6:**
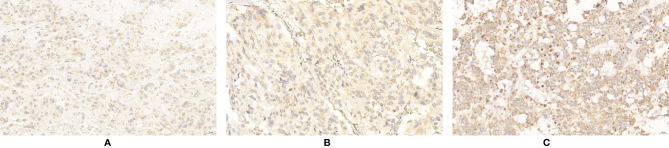
Expression of ERBB-2 in PPGLs. **(A)** Weakly positive immunostaining of ERBB-2 in PPGLs. **(B)** Intermediately positive immunostaining of ERBB-2 in PPGLs. **(C)** Strongly positive immunostaining of ERBB-2 in PPGLs.

Paraffin-embedded tumor tissues were obtained using standard surgical procedures and were selected for IHC studies. Slides were reviewed independently by two experienced pathologists, who had been blinded to clinical data, including the metastatic status. Sections with a thickness of 2.5 μm obtained from representative blocks of each tumor were deparaffinized with xylene (10 min) and gradient concentrations of alcohol (100%, 95%, and 80%) and then rehydrated. The slides were incubated at temperatures ranging from 95–98°C in EDTA buffer (ZLI-9069; ZSGB-BIO; China) with a pH of 9.0 for 20 min, followed by immersion in 3% hydrogen peroxide for 15 min. Subsequently, the sections were incubated with a primary antibody for 1 h, washed, and incubated with a secondary antibody (IB000088; ZSGB-BIO; China) at 37°C for 30 min. Finally, the antigens were detected using DAB chromogen and counterstained with hematoxylin for 2 min. A commercially available antibody against apelin (sc-293441; Santa Cruz Biotechnology Inc. USA) was used at a 1:100 dilution. Antibody against SDHB (ZM-0162; ZSGB-BIO, China) was used at a dilution of 1:1. Antibody against CHGB (MAB8868; R&D Systems, USA) was used at a dilution of 1:50. An antibody against ERBB-2 (MA5-14057; Thermo Fisher Scientific, USA) was used at a dilution of 1:400. An antibody against CNTN4 (Ab137107; Abcam, Cambridge, UK) was used at a dilution of 1:100. Antibody against SUCLG2 (sc-390818; Santa Cruz Biotechnology, Inc., USA) was used at a dilution of 1:100. For apelin, normal human breast tissue was used as the positive control. Normal human adrenal tissues were used as positive controls for SDHB and CHGB. For CNTN4 and SUCLG2, normal human kidney tissue was used as a positive control. Breast carcinoma tissue was used as a positive control for ERBB-2. Omission of the primary antibody was used as a negative control for all antibodies. This study used a semiquantitative scoring system to analyze the immunoreactivity score (IRS) results, which is described as follows: the percentage of positive (PP) tumor cells in the whole section was calculated. Subsequently, 0 points were assigned if the percentage of positive cells was 0-5%, 1 point was assigned if the percentage of positive cells was 6-25%, 2 points were assigned if the percentage of positive cells was 26-50%, 3 points were assigned if the percentage of positive cells was 51-75%, and 4 points were assigned if the percentage of positive cells was 76-100%. The staining intensity (SI) was scored as follows: 0 points for no staining, 1 point for light-yellow particles, 2 points for brown particles, and 3 points for tan particles (**Figures 1**–**6**). The final IRS results were obtained by multiplying the SI by PP. According to the final IRS analysis, the results were characterized as follows: negative staining (0 points), weak positive staining (1-4 points), intermediate positive staining (5-8 points), or strong positive staining (9-12 points). The negative staining or weak positive staining of IRS was classified as “negative expression” while IRS intermediately positive or strongly positive was classified as “positive expression” in the Kaplan-Meier analysis and the Cox proportional hazards model. The binary variables composed of them were also used to predict the metastasis state. IRS staining was scored by two independent experienced pathologists. The inconsistencies were discussed until a unified conclusion was reached.

### Statistical Analysis

All data were analyzed using SPSS 24.0. Continuous variables are expressed as mean ± standard deviation (SD), and the median and the upper and lower quartiles are presented if not normally distributed. For categorical variables, data were analyzed using chi-square (χ^2^) analysis and Fisher’s exact test. The Mann–Whitney U-test or unpaired t-test for two groups was performed to compare continuous variables. Metastasis-free survival (MFS) was defined as the time from the date of surgery to the date of metastasis confirmed by histological examination or imaging findings. Kaplan-Meier survival plots were constructed, and the log-rank test was used to evaluate survival curves. Multivariate analysis was performed using Cox proportional hazards model. The hazard ratios (HR), 95% CI, and *P* values were calculated. Metastasis was the primary outcome. A *P* value less than 0.05 was considered statistically significant.

## Results

### Patients’ Characteristics and Follow-up

Sixty patients were included in the study. The patients were divided into non-metastatic (n = 48) and metastatic (n = 12) groups. The patients were followed up until December 2020. The median follow-up time was 48.5 months (interquartile range [IQR], 35–56 months). The most common organs demonstrating metastatic progression were the lungs (4), bones (2), livers (2), kidneys (2), pancreas (1), and colon (1). For metastatic patients, the median time from the date of surgery to the identification of metastasis was 52.0 months (IQR, 32–71.25 months). One patient died of metastatic PPGL. The clinical features and follow-up data of the two groups are summarized in **Table 1**.

**Table 1:** The patients’ characteristics and follow-up data of PPGLs.

		Total	Metastatic PPGLs	Non-metastatic PPGLs	P value
PPGLs Type		60	12	48	0.367
	PCC	41	10	31	
	PGL	19	2	17	
Gender					0.843
	Male	36	8	28	
	Female	24	4	20	
Age (years)		42 (32.25, 51.5)	44 (21.25, 59.75)	41 (33, 50)	0.592
Mean diameter (cm)		4.59 (3.45, 6.64)	4.70 (3.32, 6.55)	4.48 (3.45, 6.64)	0.971
Follow up (months)		48.5 (35, 56)	52 (32, 71.25)	47.5 (35.5, 55.75)	0.517

### Expression of SUCLG2 and CNTN4 in PPGLs

Among the metastatic patients, 8 (8/12) showed weak positive staining, while 4 (4/12) showed intermediate positive staining for SUCLG2. Among the non-metastatic patients, 3 (3/48) showed negative staining, 16 (16/48) showed weak positive staining, 19 (19/48) showed intermediate positive staining, and 10 (10/48) showed strong positive staining (**Figure 1**). No significant difference was observed in SUCLG2 expression between metastatic and non-metastatic PPGLs.

For CNTN4 expression, 7 (7/12) patients showed weak positive staining, while 5 (5/12) showed intermediate positive staining in the metastatic group. In contrast, 3 (3/48) patients showed negative staining, 22 (22/48) showed weak positive staining, 21 (21/48) showed intermediate positive staining, and 2 (2/48) showed strong positive staining in the non-metastatic group (**Figure 2**). However, no significant differences were observed between them. The expression levels of these biomarkers are summarized in **Table 2**.

**Table 2:** The IHC expression of Apelin, SDHB, CHGB, ERBB-2, CNTN4 and SUCLG2 in PPGLs.

	Negative staining	Weakly positive staining	Intermediately positive staining	Strongly positive staining	P value	Percent of positive (%)	P value
**Apelin**					<0.001		<0.001
M PPGLs	6	6	0	0		7.5 (2, 20)	
NM PPGLs	0	13	33	2		70 (60, 80)	
**SDHB**					<0.001		<0.001
M PPGLs	3	8	1	0		27.5 (6.5, 50)	
NM PPGLs	0	14	32	2		80 (70, 88.75)	
**CHGB**					<0.001		0.008
M PPGLs	3	7	2	0		30 (5, 67.5)	
NM PPGLs	3	6	24	15		72.5 (40, 85)	
**ERBB-2**					0.042		0.007
M PPGLs	0	2	7	3		90 (81.25, 95)	
NM PPGLs	0	13	34	1		80 (62.5, 90)	
**CNTN-4**					0.922		0.186
M PPGLs	0	7	5	0		60 (25, 70)	
NM PPGLs	3	22	21	2		80 (32.5, 90)	
**SUCLG2**					0.13		0.212
M PPGLs	0	8	4	0		82.5 (45, 90)	
NM PPGLs	3	16	19	10		70 (50, 83.75)	

M, metastatic; NM, non-metastatic.

### Expression of Apelin, SDHB, ERBB-2, and CHGB in PPGLs

For the expression of apelin, 50% (6/12) of metastatic patients showed negative staining, and 50% (6/12) of metastatic patients showed weak positive staining. Among the non-metastatic patients, 13 (13/48) showed weakly positive staining, 33 (33/48) showed intermediately positive staining, and 2 (2/48) showed strongly positive staining for the expression of apelin. The IRS of apelin in metastatic PPGLs was significantly lower than that in non-metastatic PPGLs (χ^2^ = 30.670, P < 0.001). Similar results were observed during the comparison of the two groups using the PP method. The median PP in the metastatic group was 7.5% (2%, 20%), which was significantly lower than that in the non-metastatic group (P < 0.001). The expression of apelin is shown in **Figure 3**.

The IRS of SDHB was significantly lower in metastatic PPGLs (χ^2^ = 19.289, P < 0.001) than in non-metastatic PPGLs. The PP of metastatic PPGLs was also significantly lower than that of nonmetastatic PPGLs (P < 0.001). The expression of SDHB is shown in **Figure 4**.

For CHGB expression, 3 patients (3/12) showed negative staining, 7 (7/12) showed weakly positive staining, and 2 (2/12) showed an IRS corresponding to intermediate ely positive staining in the metastatic group. In the non-metastatic PPGL group, only 3 patients (3/48) showed negative staining for CHGB. CHGB expression was significantly reduced in metastatic PPGLs compared to that in non-metastatic PPGLs (χ^2^ = 17.235, P < 0.001). Similarly, the median PP in the metastatic group was significantly lower than that in the nonmetastatic group (P = 0.008). The expression of CHGB is shown in **Figure 5**.

ERBB-2 expression was significantly elevated in metastatic PPGLs compared to that in non-metastatic PPGLs (χ^2^ = 6.273, P = 0.042). The expression of ERBB-2 is shown in **Figure 6**. The expression levels of these biomarkers are summarized in **Table 2**.

### Importance of Apelin, SDHB, and CHGB for Differentiating Metastatic PPGLs

The specificity, sensitivity, and positive and negative prediction accuracies of apelin, SDHB, and CHGB in differentiating metastatic from non-metastatic PPGLs are summarized in **Table 3**. Apelin- and SDHB-negative expression had excellent sensitivity and negative prediction accuracy. CHGB-negative expression maintained an excellent negative prediction accuracy, with good sensitivity and specificity. The specificities of apelin and SDHB were relatively low, and the positive prediction accuracy was unsatisfactory.

**Table 3:** Specificity, sensitivity, positive and negative predictive value of apelin, SDHB and CHGB in differentiating metastatic from non-metastatic pheochromocytoma and paraganglioma.

	Specificity	Sensitivity	Positive predictive value	Negative predictive value
Apelin negative expression	72.9% (35/48)	100.0% (12/12)	48.0% (12/25)	100.0% (35/35)
SDHB negative expression	70.8% (34/48)	91.7% (11/12)	44.0% (11/25)	97.1% (34/35)
CHGB negative expression	81.3% (39/48)	83.3% (10/12)	52.6% (10/19)	95.1% (39/41)

### Predicting MFS Based on Apelin, SDHB, CHGB, and ERBB-2 Expression

**Figure 7:**
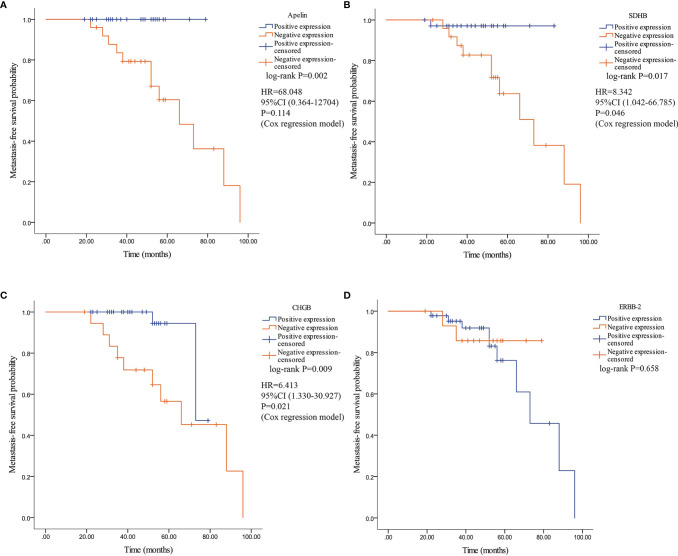
Kaplan–Meier survival curve and their related Hazard ratio in patients with PPGLs. Survival curves were plotted as graphs according to the expression of Apelin, SDHB, CHGB and ERBB-2. **(A)** Kaplan–Meier survival curve and related Hazard ratio according to the expression of apelin. **(B)** Kaplan–Meier survival curve according and related Hazard ratio to the expression of SDHB. **(C)** Kaplan–Meier survival curve and related Hazard ratio according to the expression of CHGB. **(D)** Kaplan–Meier survival curve according to the expression of ERBB-2.

Kaplan–Meier analysis revealed a significant difference in MFS between patients with PPGLs showing negative and positive expression of apelin, SDHB, and CHGB. Patients with negative expression of apelin, SDHB, and CHGB had significantly lower MFS than those with positive expression. Patients with negative ERBB-2 expression demonstrated no significant difference in MFS compared to those with positive ERBB-2 expression (**Figure 7**). Multivariate Cox analysis revealed that SDHB and CHGB levels were independently associated with MFS (**Figure 7**).

## Discussion

In patients with PPGLs, metastasis leads to a significant decrease in the survival period. Hence, early diagnosis of metastasis is of great significance. However, obtaining a definitive diagnosis for metastatic PPGLs remains difficult because malignancy can only be determined after the detection of distant metastasis in non-chromaffin tissues. Previous studies have proposed systems primarily comprising analysis of histological features to predict the metastatic potential of PPGLs, including the Pheochromocytoma of the Adrenal Gland Scaled Score (PASS) grading system and the grading system for adrenal pheochromocytoma and paraganglioma (GAPP). The PASS grading system was created by Thompson in 2002 based on 12 histological features and was the earliest grading system used to detect the potential biologically aggressive behavior of PHEOs (30). The GAPP was designed by Kimura et al. for both PHEOs and PGLs in 2014. This system was a modified version of the PASS grading system and excluded some poorly concordant histological features and added an immunohistochemical feature (Ki67 labeling index) and a biochemical parameter (catecholamine type) (31). Although previous studies have demonstrated that the reproducibility of these two systems needs to be validated in multicenter studies (1), a recent meta-analysis examining the importance of PASS and GAPP in predicting the malignancy potential of PPGLs suggested that histological analysis alone may be valuable when assessing the metastatic potential (32). To date, reliable biomarkers for distinguishing non-metastatic PPGLs from metastatic PPGLs are lacking. Recently, several immunohistochemical biomarkers that differentiate metastatic PPGLs from non-metastatic PPGLs have been increasingly identified. However, the clinical significance of these biomarkers remains uncertain, and new biomarkers are needed to identify metastatic PPGLs at an early stage.

In this study, two potential biomarkers, apelin and SUCLG2, along with four previously identified biomarkers, CNTN4, SDHB, CHGB, and ERBB-2, were studied. To our knowledge, this study is the first to detect the expression of apelin and SUCLG2 in metastatic and non-metastatic PPGLs. In our study, apelin, SDHB, and CHGB showed excellent sensitivity and negative predictive accuracy for distinguishing metastatic PPGLs from non-metastatic PPGLs. However, their positive prediction accuracy was unsatisfactory. This finding may be attributed to the small number of metastatic samples used in this study. We attempted to combine these indicators to improve their discriminatory abilities. However, the results showed that this did not improve the ability to distinguish metastatic from non-metastatic PPGLs (data not shown). The expression of apelin, SDHB, and CHGB was mostly negative or weakly positive in metastatic PPGLs, while these biomarkers primarily demonstrated intermediately positive staining or strongly positive staining in non-metastatic samples. Owing to the existence of collinearity, the combination of these indicators did not improve their prediction ability. We hope that the detection of new biomarkers will aid in a better understanding of the underlying mechanisms of metastatic PPGLs.

Recently, a number of studies have revealed an association between apelin and various cancers (15). Apelin may contribute to tumor angiogenesis and metastasis by mediating several pathways related to angiogenesis, cell migration, and cell invasion (15, 20–24). Considering that metastatic PPGLs are highly vascular tumors, we speculated that apelin may play an important role in the initiation and development of metastatic PPGLs. Interestingly, we found that apelin expression was significantly reduced in metastatic PPGLs. Half of the metastatic PPGLs demonstrated negative staining for apelin, whereas all non-metastatic PPGLs showed weakly to strongly positive staining for apelin. The risk of metastasis was significantly higher if negative apelin expression was detected in the tumor tissue. Considering the relatively small sample size of our study, a larger cohort study is required. The expression of APJ in PPGLs is worth studying in the future.

A strong association has been observed between PPGL metastasis and *SDHB* mutation (33, 34). IHC can detect *SDHB* mutations by identifying the loss of SDHB protein expression with a sensitivity and specificity of 95.0% and 81.8%, respectively (35). Notably, mutations in *SDHC* and *SDHD* may also lead to a loss of SDHB immunohistochemistry (36, 37). In our study, SDHB expression in metastatic PPGLs was significantly lower than that in non-metastatic PPGLs, which is in accordance with the results of other studies (38, 39). Kaplan–Meier analysis revealed a higher risk of metastasis in patients with negative SDHB expression. However, it is worth noting that the interpretation of SDHB IHC, especially weakly positive staining of SDHB, is still under debate (39, 40). IHC of SDHD may aid in the diagnosis of *SDHx* mutations in cases showing weakly positive staining for SDHB.

CHGB, which is encoded by *CHGB*, is a tyrosine-sulfated secretory protein expressed in different endocrine cells and neurons (12). The absence of CHGB was identified in one metastatic PHEO, while all 10 non-metastatic PHEOs demonstrated expression of CHGB, suggesting that CHGB expression may be related to malignant PHEO (41). Our study revealed a similar result: 10/12 metastatic PPGLs showed no or weakly positive staining for CHGB, while 45/48 benign PPGLs showed weakly to strongly positive staining. Traditionally, CHGB has been recognized as a house-keeping protein related to the secretory mechanisms of chromaffin cells. Currently, new roles for CHGB have been identified. Functional interaction between CHGB and the intracellular Ca^2+^ release channel, InsP3R, has been discovered (42). CHGB has also been found to regulate transcription factor expression and chromaffin cell differentiation (43, 44). Stenman et al. also found that weak or no CHGB expression detected by IHC was correlated with higher PASS scores in PPGLs. Furthermore, patients with PPGLs showing high PASS scores exhibited low preoperative plasma levels of CHGB, indicating that this non-invasive detection of the plasma levels of CHGB may have the potential to predict the IHC levels of CHGB (12).

ERBB-2, also known as HER2, belongs to the human epidermal growth factor (EGF) receptor family. ERBB-2 is associated with cell proliferation, apoptosis, and migration. The expression or amplification of ERBB-2 is correlated with various cancers, including lung, breast, ovarian, and gastric cancers (45). Yuan et al. found that the rate of metastatic PPGLs demonstrating an upregulation of the expression of *ERBB-2* was almost twice that of non-metastatic PPGLs. IHC further confirmed the overexpression of ERBB-2 in metastatic PPGLs (46). Wang et al. also found that ERBB-2 overexpression led to a significant decrease in MFS in patients with PPGLs (10). In our study, the expression of ERBB-2 was significantly elevated in metastatic PPGLs compared to that in non-metastatic PPGLs. Patients with positive ERBB-2 expression exhibited no significant difference in MFS compared to those with negative ERBB-2 expression. However, other studies have reported conflicting results. No significant difference was observed in ERBB-2 overexpression between metastatic and non-metastatic PPGLs (47). Another study revealed that ERBB-2 overexpression was not detected in metastatic or non-metastatic PPGLs (48). The differences in the criteria determined for positive staining of ERBB-2 may contribute to this inconsistency.

CNTN4, a member of the neuronal immunoglobulin superfamily, plays an important role in cell-surface interactions and can guide axonal growth. *CNTN4* mRNA was found to be overexpressed in succinate dehydrogenase (SDH)-related PHEO (5). Evenepoel et al. revealed overexpression of *CNTN4* in metastatic PPGLs. Using tissue microarrays, they detected a significantly higher frequency of samples demonstrating positive staining for CNTN4 in metastatic PPGLs (11). These results showed an association between CNTN4 and metastatic PPGLs. However, no significant difference in CNTN4 expression was found between metastatic and non-metastatic PPGLs on full slides. Our study also exhibited results similar to those of full slides. The expression levels of CNTN4 in metastatic and non-metastatic PPGLs were not consistent among studies. Future studies with larger sample sizes are needed.

PPGLs carry mutations in the genes related to the TCA cycle. However, the role of succinyl-CoA ligase (SUCL), an enzyme that participates in the TCA cycle and provides a substrate for a known PPGL-suppressor SDH, remains unclear. The expression of SUCL subunit beta SUCLG2 was found to be correlated with several tumors (49, 50). Eight germline variants in the GTP-binding domain of SUCLG2 were found in 15 patients with sporadic PPGLs. The SUCLG2 protein was absent in PPGLs with mutated SUCLG-2 and SUCLG2-deficient hPheo1 cells (28). We analyzed the expression of SUCLG2 in metastatic and non-metastatic PPGLs using IHC. No significant difference was observed between non-metastatic and metastatic PPGLs. In the future, more in-depth studies on the role of SUCLG2 in PPGLs are required.

Our study had several limitations. First, the median follow-up time was 48.5 months. Considering that metastasis may occur 20 years after initial surgery, some non-metastatic PPGLs may develop metastasis in the future. A longer follow-up period would aid in the detection of metastatic cases. Another limitation was the small size of our cohort. Third, the IHC scoring criteria used in our study may also contribute to subject bias. The inconsistent parts of the IRS scoring were discussed by the two pathologists. A third pathologist should be preferred. Multicenter studies with larger cohorts are needed in the future. Fourth, the PPGL tumor type was used as a matching condition in our study to reduce the effect of location on the expression of IHC markers. Considering that the metastatic risk of PGLs is higher than that of PHEOs, more PGLs may be selected in the non-metastatic group after the matching process, which potentially affects the true incidence of metastatic PCCs and PGLs. In addition, we did not perform genetic tests on our cohort. Recent studies have revealed the associations between genetic mutation of susceptibility genes and the development of metastasis in PPGLs. It would be meaningful to compare the results of IHC with genetic mutation data. The adding of the genetic tests would impose a positive impact on the management and prognosis of PPGLs patients.

In conclusion, the expression of apelin, CHGB, SDHB, and ERBB-2 may serve as predictive biomarkers for the diagnosis of metastatic PPGLs. Patients with negative expression of apelin, CHGB, and SDHB should be subjected to a closer postoperative follow-up procedure.

## Data Availability Statement

The original contributions presented in the study are included in the article/**Supplementary Material**. Further inquiries can be directed to the corresponding authors.

## Ethics Statement

The studies involving human participants were reviewed and approved by Medical Ethics Committee of Xiangya Hospital, Central South University. The patients/participants provided their written informed consent to participate in this study.

## Author Contributions

XG and LL designed the study. LL, YW, and DC collected and analyzed the clinical data. XG, YW, DC, YP, and XX performed the IHC analysis. YW wrote the first draft of the manuscript. XG and LL supervised the study and reviewed the manuscript. All authors contributed to the study and approved the final version.

## Funding

This research was supported by the National Natural Science Foundation of China (81902727).

## Conflict of Interest

The authors declare that the research was conducted in the absence of any commercial or financial relationships that could be construed as a potential conflict of interest.

## Publisher’s Note

All claims expressed in this article are solely those of the authors and do not necessarily represent those of their affiliated organizations, or those of the publisher, the editors and the reviewers. Any product that may be evaluated in this article, or claim that may be made by its manufacturer, is not guaranteed or endorsed by the publisher.
